# Evidence for Distinct Functions of MRE11 in *Arabidopsis* Meiosis 

**DOI:** 10.1371/journal.pone.0078760

**Published:** 2013-10-21

**Authors:** Ivica Šamanić, Juraj Simunić, Karel Riha, Jasna Puizina

**Affiliations:** 1 Department of Biology, Faculty of Science, University of Split, Split, Croatia; 2 Department of Physics, Faculty of Science, University of Split, Split, Croatia; 3 Gregor Mendel Institute of Molecular Plant Biology, Vienna, Austria; 4 Central European Institite of Technology (CEITEC), Brno, Czech Republic; National Cancer Institute, United States of America

## Abstract

The evolutionary conserved Mre11/Rad50/Nbs1 complex functions as one of the guardians of genome integrity in eukaryotes; it is required for the double-strand break repair, meiosis, DNA checkpoint, and telomere maintenance. To better understand the role of the *MRE11* gene in *Arabidopsis*, we performed comparative analysis of several *mre11* alleles with respect to genome stability and meiosis. The *mre11-4* and *mre11-2* alleles presumably produce truncated MRE11 proteins composed of the first 499 and 529 amino acids, respectively. Although the putative MRE11 truncated proteins differ only by 30 amino acids, the mutants exhibited strikingly different phenotypes in regards to growth morphology, genome stability and meiosis. While the *mre11-2* mutants are fully fertile and undergo normal meiosis, the *mre11-4* plants are sterile due to aberrant repair of meiotic DNA breaks. Structural homology analysis suggests that the T-DNA insertion in the *mre11-4* allele probably disrupted the putative RAD50 interaction and/or homodimerization domain, which is assumed to be preserved in *mre11-2* allele. Intriguingly, introgression of the *atm-2* mutant plant into the *mre11-2* background renders the double mutant infertile, a phenotype not observed in either parent line. This data indicate that MRE11 partially compensates for ATM deficiency in meiosis of *Arabidopsis*.

## Introduction

The evolutionary conserved MRN complex (MRX in yeast) is composed of Mre11, Rad50 and Nbs1 (Xrs2) proteins. The complex functions as one of the essential guardians of genome integrity by directing the processing of DNA double strand break (DSB) and is required for meiotic recombination, DSB repair via homologous recombination and end-joining reactions, DNA damage signaling, telomere maintenance and responding to stalled replication forks and resolution of DNA hairpins [1-8]. The molecular mechanism underlying these biological functions of MRX complex involves tethering DNA molecules by means of the interaction between DNA-bound MRN oligomers [9-11]. In addition, *in vitro* analyses with human and yeast proteins indicate that complex specifies 3’ to 5’ double stranded exonuclease and both double-stranded and single-stranded endonuclease activities as well as limited helicase activities [11-14]. In accordance with these biochemical activities, MRE11 plays an evolutionary conserved role in DSB resection [15]. In mice and humans the Mre11 complex is involved in DNA damage signaling and through interactions with ATM activates the DNA damage checkpoint [2,16-18]. There is no experimental evidence that MRE11 activates or interacts with ATM in plants. 

The *MRE11* gene has been identified in the genomes of all of the eukaryotes sequenced to date, including the *Arabidopsis MRE11* ortholog [19]. The homology between different Mre11 orthologs is the strongest in the N terminus which contains four conserved phosphoesterase domains, but is less pronounced in the C terminus of the protein which contains two DNA binding domains [3,13,20,21]. The N-terminal region harbors a Nbs1 interacting domain [9], while at the C-terminal region interacts with Rad50 [22]. Dynamic molecular architecture of human Mre11/Rad50/Nbs1 (MRN) consists of a globular DNA binding domain (Mre11) from which two 50-nm-long coiled coils (Rad50) protrude [9-11]. Rad50 contains Walker A and B nucleotide (NTP)-binding motifs at the N- and C- termini separated by two coiled-coil structures that can fold back on itself via zink-hook (´hinge´region) in the center of the proteins (8-10). The ´hinge´ region allows two distinct Rad50 molecules to dimerize while the ATP-binding domain on the opposite end interacts with Mre11 protein (11).The coiled coils are flexible and their apices can adopt forms of either self-association (intracomplex interaction) or intercomplex association [23]. Recent studies showed that DNA binding of human MRN complex leads to parallel orientation of the coiled coils, which prevents their intracomplex interactions and favours intercomplex associations needed for DNA tethering and biological function of MRN complex [24]. 

Originally, Mre11 was identified in yeast, *S. cerevisiae* as a gene required for early steps of meiotic recombination, namely for induction as well as for repair of meiotic DSBs. Mutational analysis of the yeast *MRE11* gene showed that its function in DSB initiation is located in the C-terminal part of the protein and is distinct from its end processing function which is associated with the N-terminal part of the protein [20,25,26]. Elucidating Mre11 function in vertebrates is hampered by the fact that null mutations in any component of the MRX complex cause embryonic lethality [27-29]. On the contrary, *Arabidopsis mre11* mutants and *rad50* mutants are viable and were shown to be sensitive to genotoxic treatment [21,30,31]. In addition, *rad50* mutation stimulates homologous intrachromatid recombination between tandem repeats in somatic cells [32]. MRE11 protein has also been implicated to play a role in an alternative DNA end-joining pathway that mediates fusion of deprotected chromosome termini [33]. 

Plants deficient for the MRE11 or RAD50 proteins are fully sterile and cytological analyses of meiosis revealed massive chromosome fragmentation during prophase I [34,35]. The fragmentation was abolished by a mutation in the *SPO11-1* topoisomerase, which is consistent with the idea that MRE11 is required for repair of the meiotic breaks [35]. In *Arabidopsis*, three T-DNA insertion mutant lines of *MRE11* gene were previously described *mre11-1*, *mre11-2* [21] and *mre11-3* [35]. While *mre11-1* and *mre11-3* mutants were both dwarfed and sterile with many developmental defects, the *mre11-2* plants displayed normal vegetative growth and fertility [21]. This indicates that C-terminal region of MRE11 is in *Arabidopsis* dispensable for fertility and meiotic recombination. However, effect of the *mre11-2* allele on meiosis has not been analyzed. 

In this study, we performed comparative characterization of *Arabidopsis* mutants harboring *mre11-2* and *mre11-4* alleles. While these alleles carry T-DNA insertions in a very similar region of the *MRE11* gene and their position differs only by 30 amino acids, they exhibited strikingly different phenotypes in regards to genome stability and meiosis. While *mre11-2* mutants are fully fertile and their meiosis is compromised only in the ATM deficient plants, the *mre11-4* mutants exhibit defective repair of meiosis DSBs and genome instability in somatic cells. This *in vivo* data indicate that the region between aa 499-529 is critical for meiotic function of the *Arabidopsis* MRE11 protein. 

## Results

### Molecular characterization of the *Arabidopsis mre11-4* allele

**Figure 1:**
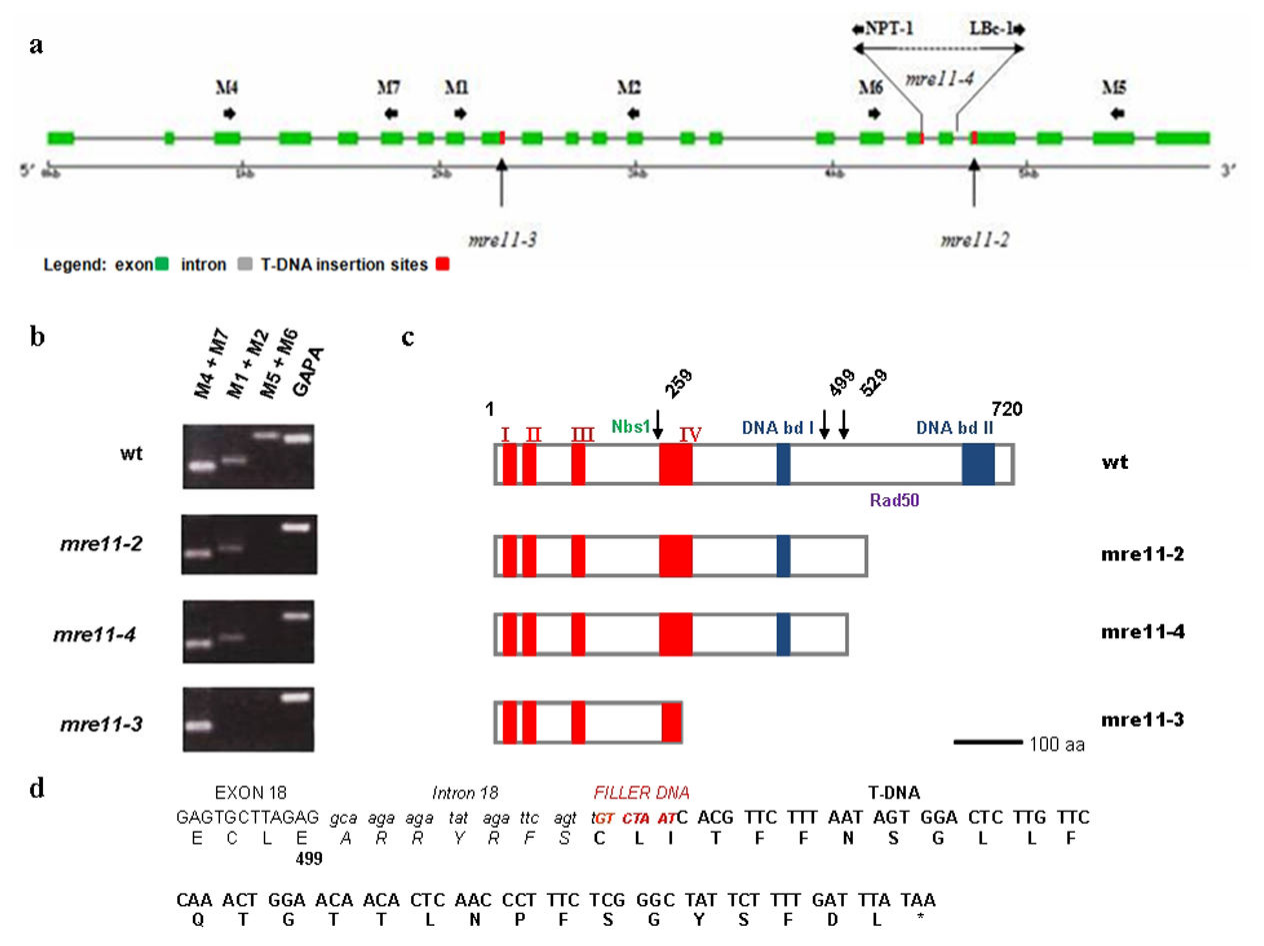
Molecular analysis and the effect of the T-DNA insertion in *mre11* mutant lines. **a**) Schematic representation of the *mre11-4* allele with the T-DNA disruption located in the 18^th^ intron (right border, NPT-1) and the left border (LBc-1) oriented toward 3´ end of the *MRE11* gene. Vertical arrows indicate the T-DNA insertion sites for *mre11-2* and *mre11-3* alleles, previously characterized [21,35]. Green boxes represent exons. *MRE11* gene specific primers are shown by short horizontal arrows. (**b**) Reverse transcriptase (RT)-PCR of *MRE11* transcripts in wild-type and three *mre11* mutants. The full-length transcripts were not produced in the three *mre11* mutants. Primers spanning different regions of *MRE11* transcripts used in the second round of RT-PCR are indicated at the top of each column. Glyceraldehyde-3-phosphate dehydrogenase A (GAPA) was used as control for cDNA amount and quality. **c**) Schematic representation of the predicted full-length MRE11 protein (wt) and putative truncated MRE11 proteins: *mre11-3* mutant lacks 461 amino acids, *mre11-4* lacks 221 amino acids and *mre11-2* lacks 191 amino acids. Arrows indicate the T-DNA disruption sites of the *MRE11* gene with respect to the full-length protein. The various putative protein domains are marked according to [8,36]; the phosphoesterase motifs (I to IV) with red boxes and two DNA binding domains (blue boxes) as well as the regions important for NBS1 and RAD50 interaction. Ideograms are drawn roughly in scale. Scale bars indicate 100 amino acids. **d**) Sequence analysis of the junction between the T-DNA and *MRE11* gene obtained via sequencing in the *mre11-4* mutants. The top line shows the genomic sequence, exon sequence is shown in uppercase letters, intron sequence is shown in lowercase italic letters, the filler DNA nucleotides are shown in small red uppercase letters and the nucleotides derived from the T-DNA insertion are shown in uppercase boldface letters. The bottom lines show the predicted amino acid sequence as a result of the T-DNA insertion. If the truncated intron 18 is not spliced out, hypothetically, 35 amino acids (ARRYRFS CLITFFNSGLLFQTGTTLNPFSGYSFDL) could be derived from the intron, filler DNA and T-DNA and form the C terminus of the predicted protein in the *mre11-4* line. The predicted STOP codon is indicated by *.

To examine the *MRE11* gene function in *Arabidopsis thaliana* we obtained a new T-DNA insertional mutant line, SALK_028450, from the Nottingham Arabidopsis Stock Centre (Nottingham, UK). The insertion was annotated within the 19th intron with the left border oriented toward the 3’end of the *MRE11* gene (Figure 1a). To precisely map the border between the T-DNA and the 5' of the gene we performed PCR reactions with primer M6 in combination with primers specific for the left or right border of the T-DNA. A specific PCR product was obtained with primers M6 and NPT-1 (data not shown). Sequence analysis of the PCR product showed that the right border of the T-DNA is located in the 18th intron, which results in a deletion of the entire 19th exon of the *MRE11* gene (Figure 1d). We refer to this mutation, which disrupts the gene in its 3' end as the *mre11-4* allele. Plants homozygous and heterozygous for the T-DNA insertion were identified by multiplex PCR with the two gene specific primers (M5 and M6) in combination with a T-DNA-specific primer (LBc-1). MRE11 ^+/-^ plants were indistinguishable from wild-type, and their self-fertilization produced homozygous mutants in the expected 3:1 ratio (26 wild-type: 8 mutants), suggesting that the *mre11-4* mutation was recessive. Details on the phenotype of *mre11-4* mutants are given below.

We next examined the effect of the T-DNA insertion on gene expression by RT-PCR and compare it with the *mre11-2* and *mre11-3* mutants. Transcription of the region upstream of the T-DNA insertion was detected in all three mutant lines (Figure 1b, primers M4+M7), while there were no transcripts from downstream region of the T-DNA insertion. This confirmed that the T-DNA insertions disrupted the synthesis of full-length *MRE11* transcripts and result in production of truncated transcripts. RT-PCR showed that *mre11-4* mutant plants similarly to *mre11-2* plants had normal levels of transcription of 5' end and middle part of the mRNA, and no expression of its 3’ end. Based on the nucleotide sequence analysis around the T-DNA insertion sites, we predicted that *mre11-4* mutants may produce hypothetical C-truncated Mre11 protein consisting of 499 amino acids (Figure 1d). Based on similar calculations that take into account only the amino acids encoded by the *MRE11* gene, it was previously shown that *mre11-3* and *mre11-2* mutants may produce hypothetical C-truncated MRE11 proteins consisting of 259 and 529 amino acids, respectively [21,35]. We were not able to confirm presence of these proteins by Western-blot analysis due to pour quality of available antibody (data not shown). 

### Comparative phenotypic and cytogenetic analysis

**Figure 2:**
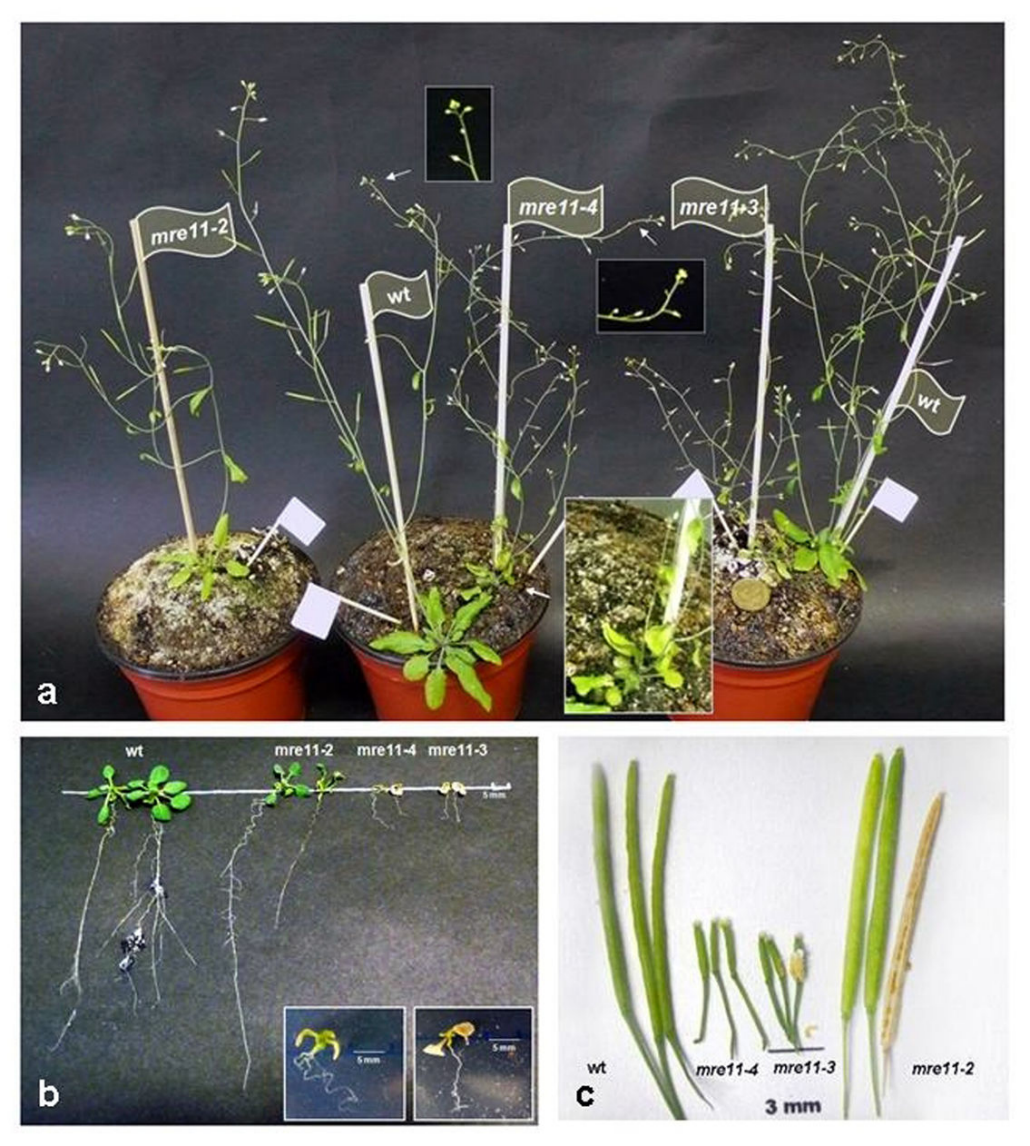
*Arabidopsis* *mre11-4* and *mre11-3* mutant alleles confer vegetative growth defects and sterility. **a**) Morphology of five weeks old *mre11* mutant plants and their comparison to wild-type plant. The arrows point at regions that are shown at higher magnification in the inserts. Coin for scale = 18 mm. **b**) Phenotypic appearance of ten-day-old wild-type (wt) and *mre11* mutant seedlings. wt and *mre11-2* mutant plants develop true leaves. In contrast, *mre11-4* and *mre11-3* (inserts) mutant plants only expand their cotyledons but do not develop true leaves and show reduced root growth. Wild-type and *mre11* mutant seeds were germinated on MS agar plates. **c**) Comparison of siliques harvested from mature wild-type and *mre11* mutants. The siliques from the *mr11-4* and *mre11-3* lines produced no seeds. *mre11-2* siliques were full (normal seed set) and were indistinguishable from wild-type. *atm-2* mutant plants are partially sterile.

To further analyze the effect of T-DNA insertion on *mre11-4* mutant growth and development, a comparative phenotypic analysis with previously characterized *mre11-2* and *mre11-3* lines was performed. In contrast to *mre11-2* plants that exhibit wilt-type appearance, plants homozygous for the *mre11-4* mutant allele are sterile and semi-dwarf with obvious morphological abnormalities (Figure 2a) and resemble *mre11-3* mutants. Rosette leaves were asymmetric and slightly upward twisted with yellow leaf margins. Microscopic analysis of *mre11-4* and *mre11-3* internal leaf structures revealed misarranged mesophyll cells with increased intercellular spaces (not shown). Vascular patterns of cotyledons were also defective showing interrupted and freely ending veins (not shown). *mre11-4* and *mre11-3* seedlings grown on vertical MS plate had reduced primary root length and secondary roots were much less developed compared with wild-type and *mre11-2* seedlings (Figure 2b). *mre11-4* as well *mre11-3* mutants produced very small seedless siliques, which contrasts with fully fertile siliques of *mre11-2* plants (Figure 2c).

**Figure 3:**
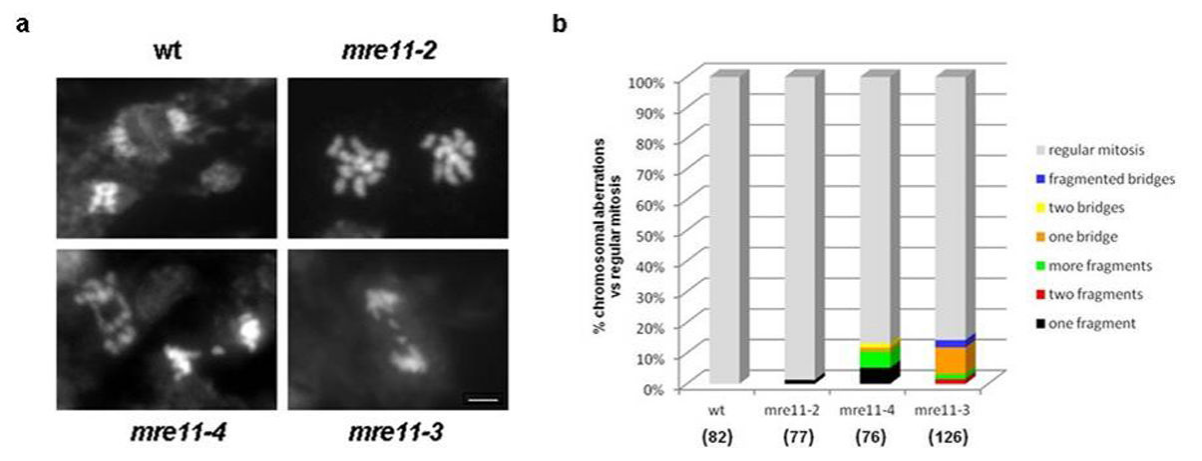
Genome instability in mitotic cells from *mre11* mutants. Anaphase spreads were prepared from pistils stained with 4’,6-diamidino-2-phenylindole (DAPI) and visualized by epifluorescence microscopy. **a**) Wild-type figure (upper left) show the phragmoplast, the cytoplasmic structure that forms at the equator of the spindle after the chromosomes have divided during the anaphase of plant mitosis. Genome instability manifested by chromosome fusions and chromosomal breaks is evident in *mre11-4* and *mre11-3* cells. Examples of *mre11-4* anaphase with two bridges and acentric fragment lagging between separating daughter nuclei are shown. Thick fragmented bridge was detected in *mre11-3* cell. Scale bar indicates 2 μm and serves all micrographs. **b**) Graphic representation recapitulating the spectrum of cytological abnormalities in mitotic cells from wild-type and *mre11* flower buds. Chromosomal aberrations in cells are classified in three categories; anaphase bridges, acentric fragments and fragmented bridges. The total number of scored mitotic cells for each genotype is indicated in parentheses.

We have previously reported that the growth defects detected in *mre11-3* mutants correlate with increased genome instability in somatic cells [35]. To investigate whether the developmental aberrations observed in *mre11-4* mutant are also associated with irregularities at cellular or chromosomal level, we performed cytogenetic analysis by comparing mitotic figures from pistil’s cells of wild-type and *mre11* mutant plants (Figure 3a). In wild-type and *mre11-2* chromosome preparations regular mitotic phases were clearly discernible. On contrary, bridged chromosomes and acentric fragments were a hallmark of *mre11-4* and *mre11-3* mitotic figures. In addition, we assessed the spectrum and frequency of chromosomal abnormalities in mitotic cells as a gauge of spontaneous genomic instability (Figure 3b). In *mre11-2* nuclei, only one acentric fragment was observed out of 77 mitotic cells, whereas *mre11-3* and *mre11-4* mutants had unstable genomes with chromosome fragmentations and fusions discovered in 13 - 14 % of the analyzed mitotic cells.

**Figure 4:**
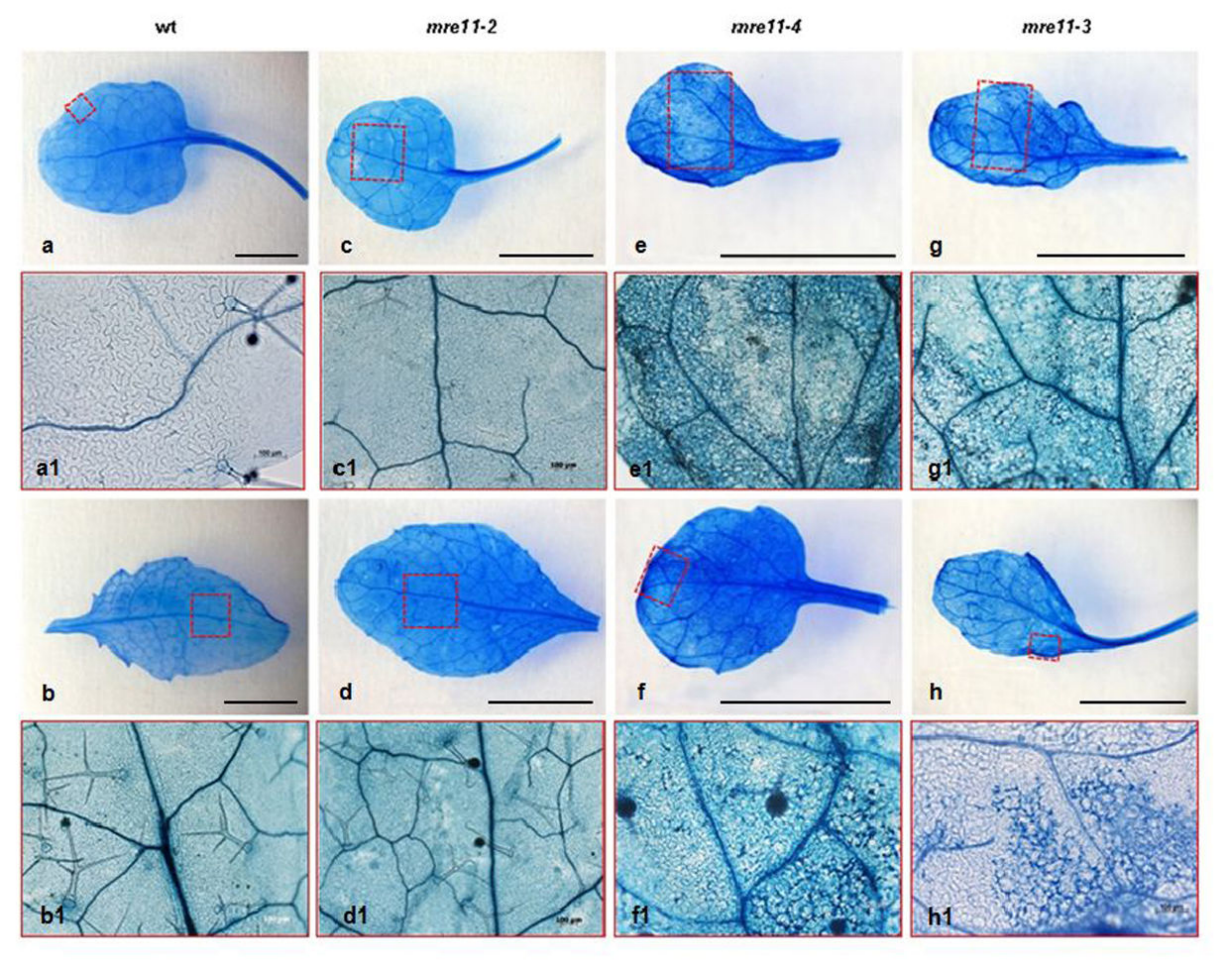
Developmental abnormalities of *mre11-4* and *mre11-3* mutants were connected with extensive spontaneous cell death. Necrotic lesions on leaves in wild-type (**a**,**b**) and three allelic series of *Arabidopsis* *mre11* mutants (**c**-**h**) were visualized by staining with trypan blue. There was extensive cell death in the leaves of the *mre11-4* (**e**,**f**) and *mre11-3* (**g**,**h**) mutant plants. In contrast, cell death was not observed in the wild-type (**a**,**b**) and *mre11-2* mutant (**c**,**d**). Leaf areas marked by red squares are shown at higher magnification below each figure (**a1-h1**). Scale bar for macroscopic leaf figures indicate 3 mm.

To determine whether the necrotic areas on *mre11-4* and *mre11-3* mutant leaves contained dead cells, trypan blue staining was performed. As shown in Figure 4, jigsaw-puzzle shaped leaf epidermis of wild type and *mre11-2* mutant plants were colorless, while there was extensive cell death in the leaves of the *mre11-4* and *mre11-3* mutant lines (Figure 4e-h1). The selected leaf surfaces of these mutants showed dark blue regions composed of irregularly shaped epidermal cells.

### Comparative analysis of meiosis

**Figure 5:**
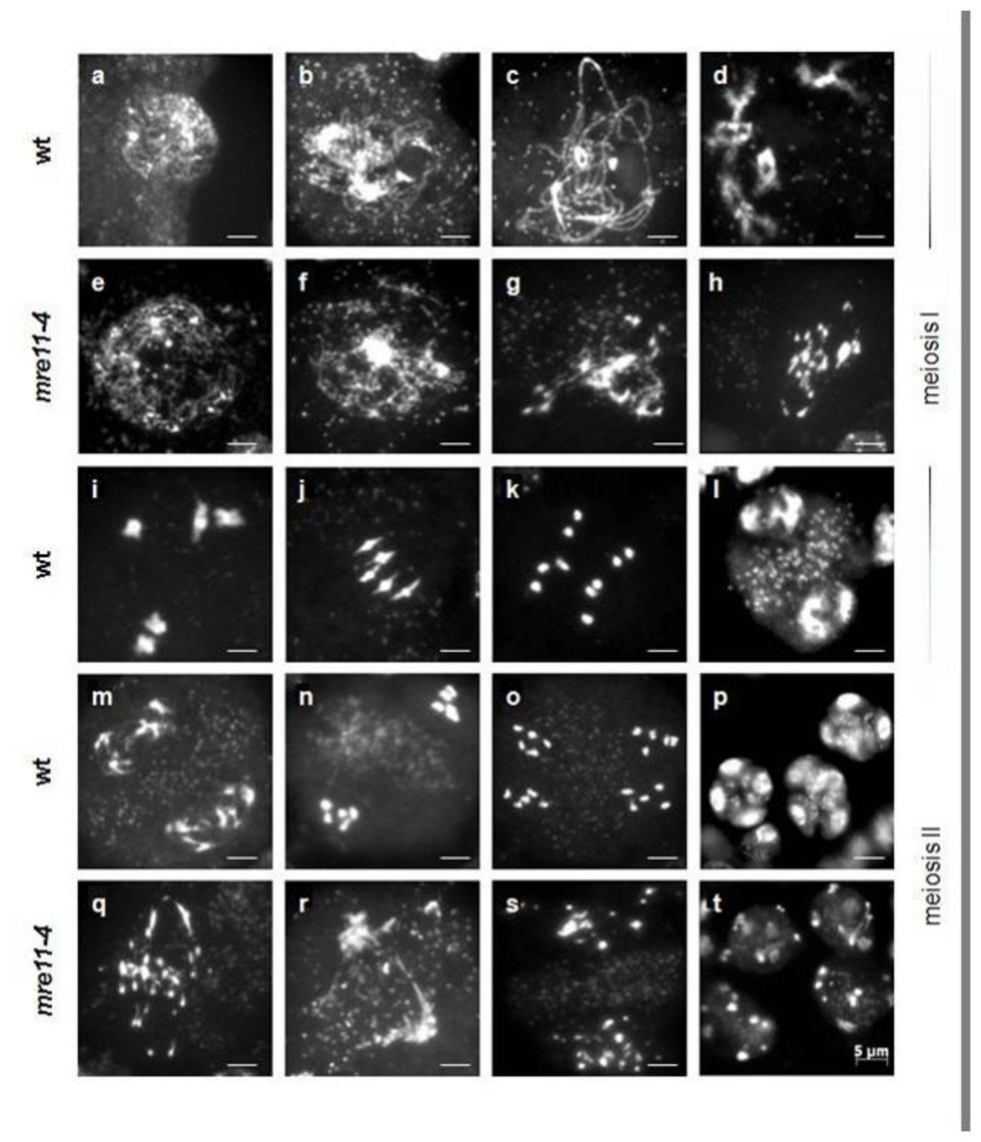
*Arabidopsis* *mre11-4* mutants exhibit dramatic chromosomal fragmentation and fusion following zygotene/ pachytene. DAPI stained chromosome spreads from pollen mother cell (PMC) meiocytes are given in (a-d) and (i-p) for wild-type, (e-h) and (q-t) for the *mre11-4* mutant. The major stages of wild-type meiosis are as follows: leptotene (a), zygotene (b), pachytene (c), diplotene (d), diakinesis (i), metaphase I (j), anaphase I (k), telophase I (l), prophase II (m), metaphase II (n), anaphase II (o), tetrads containing four microspores (p). In *mre11-4* mutant, after normal leptotene (e) and zygotene (f), all the subsequent stages were severely impaired, hence it was difficult to identify and define meiotic progression precisely. The approximate stages of *mre11-4* meiosis are as follows: late prophase I (g), post zygo-pachytene fusion/fragmentation (h,q), approximately prophase II (r), approximately anaphase II (s), polyads with variable number of microspores of different sizes (t). All micrographs have the same scale bar = 5 µm.

To investigate the origin of the sterility of *mre11-4* mutants we analyzed meiosis in pollen mother cells (PMCs). In wild-type male meiocytes chromosomes gradually condense during leptotene (Figure 5a), pair up in zygotene (Figure 5b) and synapse in pachytene (Figure 5c). Five bivalents become visible through diplotene (Figure 5d), fully condensed in diakinesis (Figure 5i) and line up in metaphase plate (Figure 5j). Homologous chromosomes move to opposing cellular poles during anaphase I (Figure 5k) and in telophase I two polar groups of chromosomes are observed (Figure 5l). During second meiotic division sister chromatids separate to finally give the four haploid microspores (Figure 5m-p).

In *mre11-4* mutants regular prophase was absent and all the subsequent stages of meiosis were severely impaired. After normal leptotene (Figure 5e), fragmented chromosome threads appeared at the mid-prophase stage that corresponds to the wild-type zygotene-pachytene (Figure 5f). A typical looped ribbon-like structure, normally present in wild-type pachytene, was never observed in *mre11-4* mutants, suggesting a failure to synapse homologous chromosomes in the absence of MRE11 function. Chromosome fragmentation became more visible as chromatin continued to condense in the subsequent stages of post zygo-pachytene and varying sizes and numbers of chromosome fragments, but no regular bivalents were observed in all PMCs (Figures 5g-h). Second meiotic division was identified based on the appearance of the typical organellar band in the middle of the PMCs. In spite of severe chromosomal fragmentation, meiosis progressed into meiosis II (Figures 5 r-s) and finished with polyads, containing microspores with unequal amounts of DNA (Figure 5t). This phenotype is comparable with meiotic defects observed in *mre11-3* mutants [35].

**Figure 6:**
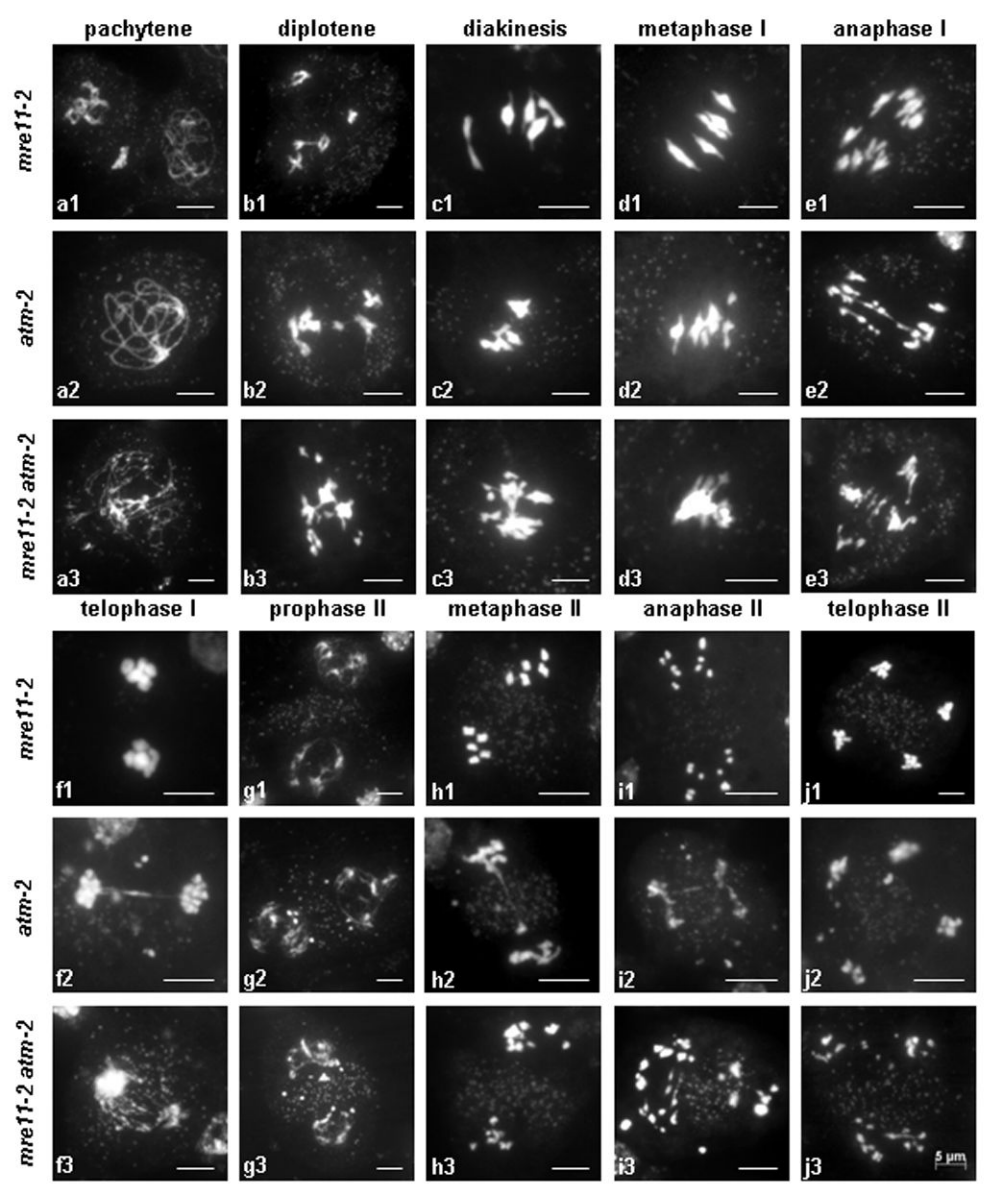
Meiotic phenotypes of *mre11-2*, *atm-2* and *mre11-2* *atm-2* double mutants. *atm-2* prophase proceeds to pachytene (**a2**), when fully synapsed chromosomes are visible. First abnormalities were bridges between bivalents in diplotene (**b2**) and multiple lumping of bivalents together in diakinesis (**c2**). Metaphase I (**d2**) shows univalent far from aligned bivalents at equatorial plane. Chromosome bridges formed between the separating groups of anaphase I chromosomes (**e2**) are accompanied by lagging acentric fragments and chromatid bridges between the two nuclei (**f2**). Subsequent stages of *atm-2* meiosis II also display chromosome fragmentation and chromatin bridging across the organelle band (**g2-i2**). In telophase II several discrete DAPI-stained fragments are visible outside of the four groups of chromosomes (**j2**). Meiosis in *mre11-2* *atm-2* mutant is seriously impaired at all post-leptotene stages (**a3**) when long tracts of unpaired chromosomes were observed. The most common cytological phenomenon is the chromosome stickiness (**b3**-**d3**). At anaphase I (**e3**) sticky chromosomes lagged behind stretched chromosomes which began to separate to opposing poles. At telophase I (**f3**) multiple chromosome bridges were stretched between two groups of chromosomes at poles. The irregularities of meiosis II include intensively stained chromatin mass between partially decondensed dyad nuclei (**g3**); uneven distribution of chromosomes at metaphase II (**h3**); chromatin bridges across the organelle band in anaphase II (**i3**) and chromosomal fragments excluded from four distinct groups of chromosomes at telophase II (**j3**). All micrographs have the same scale bar = 5 µm.

**Figure 7:**
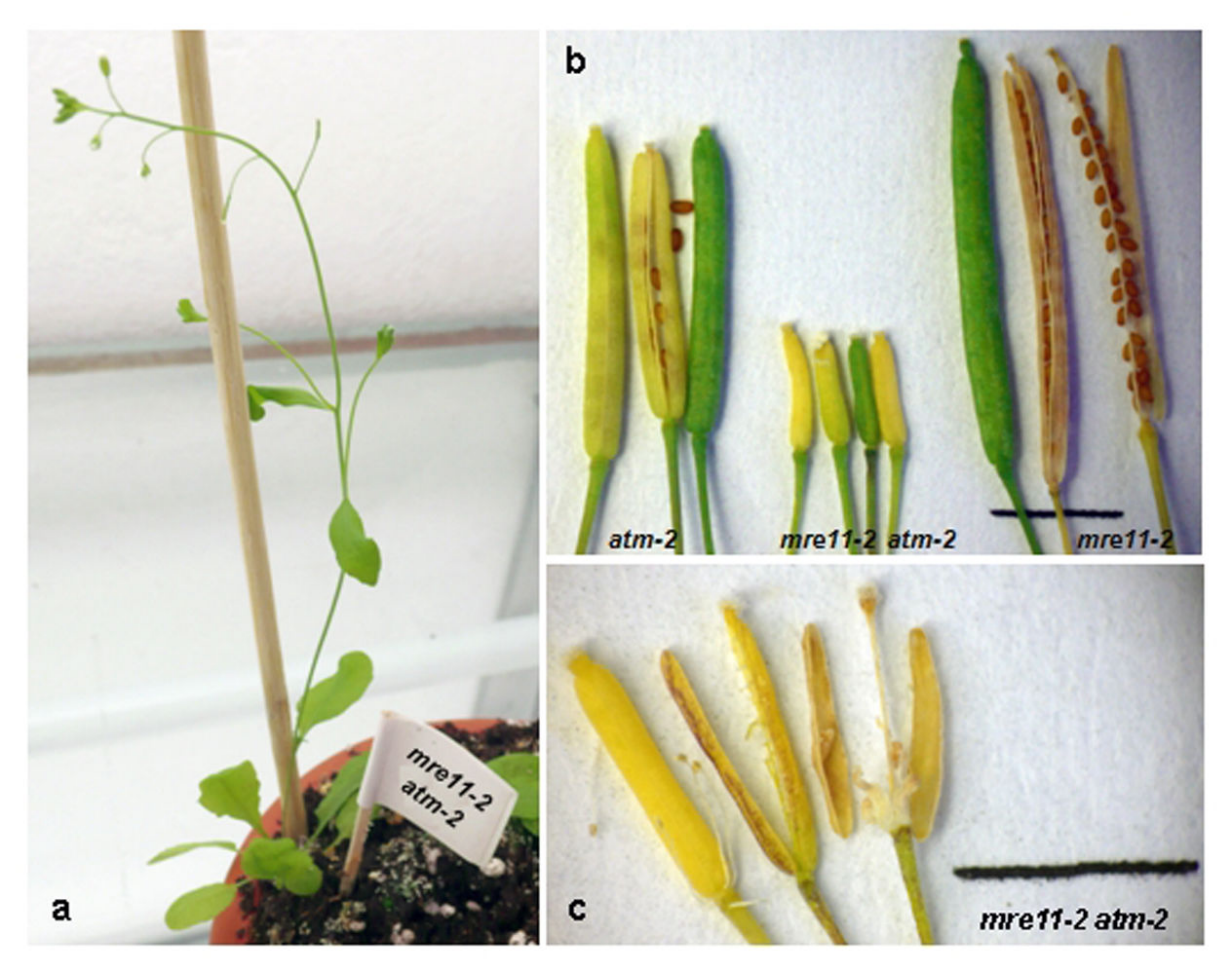
*Arabidopsis* *mre11-2* *atm-2* double mutant plants form empty siliques and are completely sterile. **a**) Morphology of five weeks old *mre11-2* *atm-2* double mutant plant. **b**) The siliques from the *mre11-2* *atm-2* double mutant line produced no seeds, *mre11-2* siliques had normal seed set and *atm-2* mutant plants were partially sterile. **c**) Empty siliques of *mre11-2* *atm-2* double mutants - larger magnification.

Unlike *mre11-4* mutant plants which are completely sterile, homozygous *mre11-2* mutants are fully fertile [21] and we did not detect any cytological abnormalities in meiosis (Figures 6 a-l). Although *mre11-2* mutants have phenotypically normal appearance, they are still sensitive to DNA damage [21] indicating that function of this protein is compromised. MRE11 acts in DNA damage signaling in conjunction with ATM. It has been reported that function of ATM is in meiosis partially redundant with ATR and NBS1. To investigate whether MRE11 acts redundantly with ATM, we generated *mre11-2 atm-2* double mutant. The *atm-2* single mutants exhibit drastically reduced fertility, but still produce reduced seed number [16]. In contrast, *mre11-2 atm-2* double mutants are completely sterile (Figure 7) indicating severely impaired meiosis (Figure 6).

Cytogenetic analysis in *atm-2* mutants showed that early stages of prophase I occurred normally up to pachytene, when homologous chromosomes were completely aligned along their length (Figure 6 a2). The first abnormalities in *atm-2* meiosis become visible during the diplotene and diakinesis when chiasmata between bivalents (Figure 6 b2) and entangled multivalents (Figure 6 c2) were observed, suggesting that nonhomologous pairing and synapsis could occur during *atm-2* meiosis. At metaphase I the unpaired chromosomes are displaced from bivalents that are aligned at equatorial plane (Figure 6 d2). However, the most obvious and frequent anomalies are chromosome fragmentation and abnormal stretches of chromosomes observed during anaphase-telophase I transition (Figure 6, e2 and f2). Bridges between univalents or chromatids accompanied by fragments were also present in meiosis II (Figure 6, g2- i2). At the end of *atm-2* meiosis in telophase II several discrete fragments are seen outside of the four groups of chromosomes (Figure 6 j2). A similar meiotic phenotype was previously described for the *Arabidopsis atm-1* mutant line [16].

Chromosome integrity in male meiocytes of *mre11-2 atm2* double mutant was strongly affected; starting from late zygotene/early pachytene-like stages when homologous chromosomes fail to synapse and become extensively fragmented (Figure 6a3). During prophase I chromosomes were clumped together into groups of various sizes. Chromosome stickiness ranged from small aggregations that they might represent fragments of chromosomes (Figure 6b3) to multiple interconnected chromosomes (Figure 6 c3) and compact chromatin mass on the metaphase plate involving the entire chromosome complement (Figure 6 d3). The stickiness of chromatin caused impaired chromosome segregation in the first meiotic division (Figure 6, e3 and f3). Dyad stage meiocytes show several DAPI bright chromatin bodies lagged in the region of organelle band between the reforming nuclei (Figure 6 g3). At metaphase II unbalanced chromosome groups with distinctly sized units are evident (Figure 6 h3). Additional chromosome fragments appeared during separation in anaphase II (Figure 6 j3). The meiotic products of the second division were distributed into four groups of varying sizes (Figure 6 j3). Eventually, unequal nuclei reformed to produce equivalent of the tetrads with excluded laggard chromosomal bodies that did not contribute to microspores formation. This indicates that MRE11 partially compensates for ATM deficiency in meiosis and that *mre11-2* plants are impaired in this function. 

### *In silico* analysis of the predicted truncated MRE11 proteins

**Figure 8:**
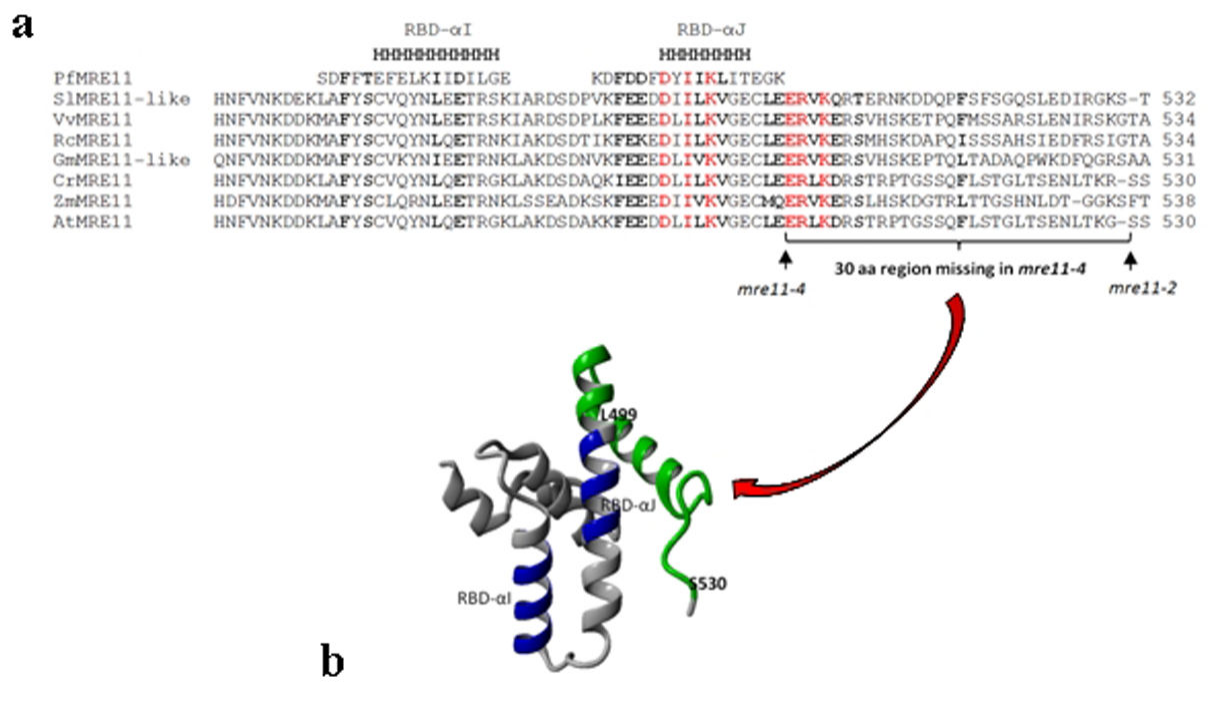
The putative Rad50 binding domain of AtMRE11protein in several plant species and its predicted three dimensional structure. **a**) Alignment between partial MRE11 sequences of *Pyrococcus furiosus* (Pf), *Arabidopsis thaliana* (At), *Solanum lycopersicum* (Sl), *Vitis vinifera* (Vv), *Ricinus communis* (Rc), *Glicinia* *max* (Gm), *Capsella rubella* (Cr) and Zea mays (Zm). RBD-αI and RBD-αJ helices that make the RAD50 binding domain are indicated. **b**) Predicted three dimensional structure of the partial AtMRE11 protein. RBD-αI and RBD-αJ helices are marked in blue. 30 amino acids, which differ the putative MRE11 proteins coded by *mre11-4* allele from *mre11-2* allele, L499 to S530 are marked in green.

To better understand the functional differences between the *mre11-2* and *mre11-4* alleles, we searched for structural homology in the region that is supposed to be truncated in the *mre11-4* allele, but was presumably retained in the *mre11-2* line (Figure 8a). Interestingly, this region is positioned immediately downstream of the recently identified Mre11-Rad50 binding domains (RBDs) in *Pyrococcus furiosus* [36]. Multiple sequence alignment of MRE11 fragment from seven plant species shows that the *mre11-4* deletion partially overlaps with a region that is highly conserved in plants (Figure 8a). 

In order to obtain a more detailed information of the protein region affected by the *mre11-4* deletion, we used the homology based prediction algorithm to get the three dimensional structure of affected region of MRE11. Alignment between the partial RBD sequences of *P. furiosus* and seven different plant species shows the positions of the helices RBD-αI and RBD-αJ. Sequence similarity between *P. furiosus* and *A. thaliana* for RBD-αI and RBD-αJ was 37.5% and 50% respectively. The second RBD helix is placed adjacent to the *mre11-4* deletion, which may affect the function of this domain and the MRE11 protein (Figure 8 b).

## Discussion

In this study, we compared two mre11 alleles, *mre11-4* and *mre11-2*, that presumably produce putative truncated MRE11 proteins composed of the first 499 and 529 amino acid residues, respectively. However, despite this relatively small length difference in the C-terminus, the phenotypic differences between *mre11-2* and *mre11-4* mutant lines are striking: *mre11-2* mutants display normal phenotype and fertility while *mre11-4* plants were sterile with many developmental defects. 

The chromosomal instability observed in *mre11-3* and *mre11-4* mutants lead to accumulation of DNA damage and consequently to increased cell death, which has been confirmed by trypan blue staining and reduction in the number of leaf cells. These results are in agreement with immunolocalization of γ-H2AX foci in in *rad50* and *mre11* root tips and reduced cell division in those cells [37]. This suggests that MRE11 function is severely compromised in these mutants. In contrast, *mre11-2* plants look phenotypically normal arguing that this allele produces a truncated peptide that retains at least partial functionality, which may depend on aa residues 499-529. This region is very close to the Mre11 Rad50 binding domains (RBD) recently identified in *Pyrococcus furiosus* [36]. We suggest that T-DNA insertion in the *mre11-4* allele has probably disrupted the putative Rad50 interaction and/or homodimerization domain, which is assumed to remain preserved in *mre11-2* allele. The sequence conservation around the insertion site of the *mre11-4* allele supports the hypothesis of the functional importance of the deleted region. 

Using conditional mouse cell lines that either abrogate nuclease activities or inactivate the entire MRN complex, the essential function of MRN has been associated with the nuclease activity [15]. Lack of the nuclease activity causes phenotypes indistinguishable from the null allele, including early embryonic lethality and dramatic genomic instability. Although the *mre11-4* allele could have preserved all the predicted nuclease domains, nevertheless phenotypically it is indistinguishable from *mre11-3* mutant, which harbors disruption in the putative nuclease domain. According to our *in silico* model, deletion of RBD could have equally deleterious consequences for function of MRN complex as mutations in the nuclease domains. However, one must take into consideration the possibility that the *mre11-4* could represent a 'null' allele, thus expressing no protein at all. Sterility and morphological resemblance between the *mre11-3* mutants, which are assumed to be ‘null’ [35], and *mre11-4* mutants could, possibly, suggest such an alternative interpretation of the data.

We demonstrated that *mre11-4* mutants had an aberrant meiotic phenotype, very similar to the meiotic phenotype of *mre11-3* mutants, which was characterized by severely fragmented chromosomes – consequence of unrepaired and misrepaired SPO11 induced DSBs [35]. The experimental evidence gathered in a number of organisms demonstrates that the Mre11 complex is required for processing of Spo11 induced meiotic DSBs, and permits homolog pairing, recombination and bivalent formation [38,39]. Recent studies suggest that Mre11 endo/exonuclease activities and Exonuclease 1 (Exo1) are required for removing Spo11- oligonucleotides from DSB ends [40] and for subsequent bidirectional resection of DSBs [41]. Thus the *mre11-3* and *mre11-4* alleles are deficient in repair of meiotic breaks. 

In contrast, the *mre11-2* allele that lacks 191 terminal amino acids is fully proficient in meiotic repair demonstrating that the C-terminus not be required for DSB repair in *Arabidopsis*. Similarly, in mammals, MRE11^ALTD1/ATLD1^ mutation caused by C-terminal 75-amino acid deletion is not associated with meiotic abnormalities in mice [42]. Although in budding yeast is the C-terminal part of the Mre11 protein required for DSB induction in meiosis, studies with separation of function mutants revealed that N-terminus is crucial for the DSB processing and repair [20,26]. We have previously demonstrated that MRE11 is not required for DSB induction in *Arabidopsis* [35], which is corroborated by the lack of any apparent meiotic defects in *mre11-2* mutants. Nevertheless, we found that that *mre11-2* causes in ATM deficient plants infertility and meiotic phenotype characterized by lack of chromosome pairing and defects in meiotic double strand break repair, suggesting that MRE11 protein and ATM kinase have a redundant meiotic function that is distinct from DSB repair. A similar genetic interaction has also been observed in *nbs1-1 atm-3* double mutants, which are sterile, although *nbs1-1* mutants are fertile and *atm-3* mutants semi-fertile, thus suggesting that *Arabidopsis* ATM kinase plays synergistical role with NBS1 in the control of meiotic events [43]. 

Hypersensitivity of *mre11-2* line to genotoxic treatments [21] suggests that C-terminus of the MRE11 protein is involved in DNA damage signaling/and or checkpoint activation, most-likely through interaction with NBS-1 and subsequent ATM/ATR activation. This assumption comes from the Mre11 structure defined by the X-ray crystallography, which shows that C-terminal domain close to the hydrophobic region is important for protein-protein interactions [8,11,44]. It was assumed that C-terminal truncation of Mre11 reduces protein interactions within the MRN complex as well as its interactions with other damage-response proteins, including ATM kinase. 

New research suggests that the Mre11 C-terminus is playing a previously unknown role in human somatic dividing cells. It has been shown that Mre11 C-terminus interacts with CDK2 and governs the overall levels and the phosphorylation status of the CtIP protein and its interaction with BRCA1. This oligomeric protein complex controls the capacity of cells to initiate resection at DSBs and restricts the use of homologous recombination to cell cycle phases when sister chromatids are present and its function does not require ATM activation [45]. Although the significance of the mammalian protein CtIP in meiosis has not yet been elucidated, based on the phenotype of *com1-1* mutant line, an *Arabidopis* homologue of the yeast Com1/Sae2 and closely related to the mammalian CtIP, it has been predicted that CtIP in *Arabidopsis* is required for meiotic DSB repair [46]. The confirmation of such genetic interaction would probably explain the complete sterility of double *mre11-2 atm-2* mutant line.

### Conclusion

The results of comparative molecular, cytogenetic and morphological characterization were showed that two *Arabidopsis* mutants harboring *mre11-2* and *mre11-4* alleles be strikingly different with regard to genome stability and meiosis. The structural analysis of the region surrounding the T-DNA insertion suggests that the region between aa 499-529, which probably represents the part of RAD50 interaction and/or homodimerization domain, is critical for the meiotic double strand break repair function of the *Arabidopsis* MRE11 protein. The fact that meiosis of *mre11-2* mutants is compromised only in the ATM deficient plants, suggest that C-terminus of the MRE11 protein, which was previously assumed to be dispensable for *Arabidopsis* meiosis, is associated with another, currently unknown, meiotic function of MRE11 in *Arabidopsis*, probably related to DNA damage signaling.

## Material and Methods

### *Arabidopsis* lines and growth conditions

Seeds of the *mre11-4 Arabidopsis thaliana* SALK_028450 line, ecotype Columbia (Col-0), were obtained from the Nottingham Arabidopsis Stock Centre (Nottingham, UK). Because *mre11-4* mutants are sterile, the *mre11-4* allele was maintained via self-fertilization of heterozygous plants. Double mutants were produced by crossing the *atmre11-2* mutants into the *atatm-2* background and screening subsequent generations. All plants were cultivated in a growth chamber under long-day condition (16-h light/8-h dark) at 23 °C, on a mixture of peat (Type 3 special, Gebr. Brill Substrate, Germany) and a silicaceous material of volcanic origin (Agrilit 3, Perlite Italiana, Italy). In order to break seed dormancy and allow coordinated germination, seeds were placed on moist filter paper for 48-h at 4 °C in Petri dishes wrapped with parafilm.

For comparative phenotypic analysis of wild-type and *mre11* seedlings, seeds were sown on medium (pH 5.8) containing Murashige and Skoog (MS) basal salt mixture (4.39 g/L, Sigma) plus Gamborg`s B-5 Basal Salt Mixture (3.1g/L, Sigma), MES monohydrate (0.5 g / L, 4-Morpholineethanesulfonic acid monohydrate, Fluka), sucrose (10 g/L) and agar (6 g/L, Plant agar, Duchefa, Biochemie). Before planting, *Arabidopsis* seeds were surface sterilized with 70% ethanol for 1 min, then with bleach solution (6.15% sodium hypochlorite solution plus 0.1% Triton X-100) for 7 min, and subsequently rinsed by sterile distilled water 5 times, and then kept in the dark for 48-h at 4° for stratification. The plates were then transferred to a 23 °C chamber for ten days. 

### DNA analyses

Plant genomic DNA was extracted from 0.05-0.2 g *A. thaliana* fresh leaf tissue using the hexadecyltrimethylammonium bromide (CTAB) extraction buffer [47]. Plants were genotyped by PCR with two gene specific primers flanking insertion site and with a primer specific for left border of the T-DNA. Primers M5 (5'-CGTCATCGTCTTTG-CTACTGAGTA-3') and M6 (5'-ATGGAGATCCTTCCAGTTAACGAT-3') and LBc-1 (5'-TGGACCGCTTGCTGCAACTCT-3') were used for the MRE11 locus, resulting in 1329-bp and ~1- kb products for the wild-type and *mre11-4* alleles. As the *atmre11-2 atatm-2* double mutant is completely sterile, we PCR- screened F_2_ offspring for homozygous insertion mre11-2^-/-^ [gene-specific primers M5 and M6 plus the T-DNA specific primer JL-202 (5'-CATTTT ATAATAACGCTGCGGACATCTAC-3'] and homozygous insertion atm-2^-/-^ [gene-specific primers AtmF19 (5'-CTTGCCTCCCAGAAAAATGTTATT-3') and AtmR19 (5'-ACACTTCCTCT-AAACTCAACTATCAGACG-3') plus T-DNA specific primer LBc-1. Two 650-bp and 850-bp products were generated from the *mre11-2* allele and 650-bp product from the *atm-2* allele. Purified DNA sample (10 ng/μL) was added to PCR mixture (final volume of 20 μL) that contained 10x PCR buffer minus Mg (1x), MgCl_2_ (1.5 mM), dNTP mixture (0.2 mM), 0.5 μM concentrations of each of the primers and 0.05 U/ μL of recombinant *Taq* DNA polymerase (Invitrogen). PCR condition for the ATM locus were as follows: 94°C for 2 min, followed by 10 cycles with a decrease in annealing temperature (94°C for 15 s, 65 to 0.5°C per cycle for 15 s, and 68°C for 2 min), followed by 35 cycles (94°C for 15 s, 60°C for 15 s, and 68°C for 2 min) and final extension step at 68°C for 7 min. The conditions of *MRE11*-PCR were as follows: 94°C for 4 min; 35 cycles of 94°C for 15 s, 60°C for 30 s, and 72°C for 60 s; and 72°C for 7 min.

### RNA extraction and RT-PCR

Total RNA was extracted from young inflorescences using the TriReagent solution (Sigma, St. Louis, MO). RNA samples were DNase-treated (DNase Set; Qiagen) and quantified. First strand *MRE11* and *GAPA* cDNAs were synthesized as follows: 5 pmol of primers M7 (5'-ACACCAGAACCACCAAGAACCAT-3'), M2 (5'-CCAATGGGAGTTTGATCTCTGA-3'), M5, LBc-1 and GAPA-2 (5'- CAACTCTCTGTGAGTAACCCCAT-3') were incubated with 2 μg of total RNA at 70°C for 5 min. Reverse transcription was performed with M-MLV (H^-^) reverse transcriptase (8 U/ μL, RevertAid TM *H Minus* Reverse Transcriptase, Fermentas) in a 25 μL reaction volume at 37 °C for 50 min. *MRE11* fragments were amplified from cDNA pools by PCR with following gene-specific primer combinations: M4 plus M7; M1 (5'-CCAATGGATGAGGCC-TGAAGTT-3') plus M2; M5 plus M6. Positions of the primers relative to the *MRE11* gene are shown schematically in Figure 1a. PCR thermal cycling conditions were as follows: 4 minutes at 94°C, 25 cycles of 15 seconds at 94°C, 30 seconds at 60°C and 1 minute at 72°C, followed by 7 minutes at 72°C. Control RT-PCR with GAPA2 and GAPA3 (5´-TCTTCTCCCTTGGAAGGAGCT-3´) primers specific for glyceraldehyde-3-phosphate dehydrogenase A (GAPA) were performed for 20 cycles. 

### Analysis of meiotic and mitotic chromosomes

Inflorescences of both the wild type and mutants were harvested and fixed in Carnoy`s solution (ethanol:glacial acetic acid, 3:1) and stored at 4°C. Mitotic figures were obtained from pistils of unopened floral buds. Meiotic figures were prepared from anthers of young floral buds (~ 0.5 mm in diameter). After washing the inflorescences in water and 0.01M citrate buffer (pH 4.7), pistils or anthers were dissected under the stereo microscope and transferred to an enzyme mixture containing 0.5% (w/v) cellulase Onozuka R-10 (Serva, Heidelberg, Germany) and 0.5% (w/v) pectolyase (Sigma) in 0.01 M citrate buffer. The enzyme mixture was replaced by citrate buffer after ~45 min of incubation at 37°C in a moist chamber. Citrate buffer was removed and pistils or anthers were squeezed by a pinhead on the glass slide in a drop of 45% acetic acid. After freezing slides at −80°C, coverslip was removed and slides were air-dried. Slides were stained with 2mg/mL of DAPI and mounted in Vectashield antifade solution (Vector Laboratories, Burlingame, CA). Identification of meiotic stages was performed according to [48,49]. Images were captured under a Zeiss Axio-imager M1 Epifluorescence and Brightfield Microscope with a high-resolution microscopy camera (Carl Zeiss AxioCam MR Rev3) using Axio Vision Rel. 4.7 softer (Karl Zeiss, Vienna, Austria).

### Cell death assay

Necrotic lesions on leaves were detected by lactophenol-trypan blue staining [50]. Stain solution was prepared by mixing 8g of phenol, 8 mL of glycerol, 8 mL of lactic acid, 8 mL of distilled water and 8g of trypan blue (Sigma).Whole leaves were boiled for approximately 1 min in the stain solution, incubated overnight at room temperature and then decolorized in chloral hydrate (2,5g/ mL). Trypan blue-stained areas were examined under a microscope. 

### *In silico* analysis of the predicted truncated MRE11 proteins

Gene structure schematic diagram is drawn by Gene Structure Display Server (GSDS) available at the Center for Bioinformatics (CBI) on Peking University (http://gsds.cbi.pku.edu.cn/) [51]. The prediction of three-dimensional structure of *A. thaliana* MRE11 fragment was done using I-TASSER server (http://zhanglab.ccmb.med.umich.edu/I-TASSER/). As an additional restraint for homology based modeling, *A. thaliana* MRE11 protein was aligned with RAD50 binding domain of the Mre11 protein of *Pyrococcus furiosus* [36], which structure was retrieved from PDB database (PDB ID: 3QKU, 3QKS). All sequence alignments were done by Muscle algorithm within MEGA5 software package [52].
